# Critical illness-related corticosteroid insufficiency in patients with low cardiac output syndrome after cardiac surgery

**DOI:** 10.1186/2197-425X-3-S1-A546

**Published:** 2015-10-01

**Authors:** JY Lim, SH Jung, JB Kim, SJ Choo, CH Chung, JW Lee

**Affiliations:** Asan Medical Center, University of Ulsan College of Medicine, Seoul, Republic of Korea

## Introduction

Low cardiac output syndrome (LCOS) which features classic symptoms of hypotension, tachycardia, oliguria and poor peripheral perfusion after cardiac surgery is not rare in the immediate postoperative period, and usually requires high dose of inotropes and volume replacement. In this setting of critical illness and stress, Critical Illness-Related Corticosteroid Insufficiency (CIRCI) may occur and aggravate hemodynamic instability. However, lack of evidence or guideline exists regarding diagnostic criteria of CIRCI in LCOS or use of steroids.

## Objectives

We aimed to investigate clinical features of CIRCI in patients with LCOS after cardiac surgery and assessed the efficacy of steroid use in this subset of patients.

## Methods

We retrospectively reviewed the patients who were suspected as combined CIRCI in the setting of LCOS after cardiac surgery between February 2010 and September 2014. Diagnosis of CIRCI was made by a delta total serum cortisol of < 9µg/dL after ACTH 250 µg administration or a random total cortisol of < 10 µg/dL. Patients who were exposed to steroids before surgery for any medical causes were excluded.

## Results

28 patients underwent adrenocorticotropic hormone (ACTH) stimulation test in suspicion of CIRCI. Among them, 20 patients met the diagnostic criteria. Mean age was 63.8 ± 12.4 years. Majority of patients (80%) underwent valvular surgeries or pericardiectomy. Patients who were diagnosed with CIRCI (+) showed higher Sequential Organ Failure Assessment (SOFA) score at the time of diagnosis compared to CIRCI (-) patients (16.1 ± 2.3 versus 11.4 ± 3.5, p = 0.001) and higher total bilirubin level (11.7 ± 9.2 µmol/L versus 4.9 ± 4.3 µmol/L, p = 0.015). Glasgow Coma Scale (GCS) score was lower in CIRCI (+) group (7.9 ± 3.9 versus 11.2 ± 3.2, p = 0.048). 3 patients (10.7%) died only in CIRCI (+) group without statistical significance. Among CIRCI (+) patients, only 6 patients (30%) received glucocorticoid therapy at surgeon's discretion. Initial dose of steroid therapy varied from 50 to 240 mg per day. Main reason not to use steroid to the other patients was the concern of infection. Mean blood pressure was elevated by the average of 22.2 ± 8.7mmHg after steroid therapy and duration of inotropic support was significantly shorter in steroid therapy group compared to non-steroid therapy group (4.1 ± 2.3 versus 30 ± 22.8 days, p = 0.001). Infection developed in 3 patients (15%) only in non-steroid therapy group without significant difference.

## Conclusions

Critical Illness-Related Corticosteroid Insufficiency should be suspected in patients with Low cardiac output syndrome after cardiac surgery especially when patients shows signs of persistent multi-organ failure such as high SOFA score or total bilirubin level. Glucocorticoid replacement therapy may be considered in patients with Critical Illness-Related Corticosteroid Insufficiency after cardiac surgery to reduce the use of inotropes without increasing additional infection risk.

